# Supplementary material for: Longitudinal host transcriptional responses to SARS-CoV-2 infection in adults with extremely high viral load
Source: PLoS One. 2025 Jan 16;20(1):e0317033. doi: 10.1371/journal.pone.0317033 (PMC11737797; doi:10.1371/journal.pone.0317033)
Supplement: S1 Table — (DOCX) [file pone.0317033.s001.docx]

TABLE S1. Demographic and Visit Characteristics by Initial Matched Groups

| \|  \| Extremely high viral load cases \| Low viral load cases \| Negative for SARS-CoV-2 \|  \| \| --- \| --- \| --- \| --- \| --- \| \|  \| (n = 10) \| (n = 10) \| (n = 10) \| p-value \| |
| --- | --- | --- | --- | --- | --- | --- | --- | --- | --- | --- |
| \| **Age^a^**, years \| 51.5  (22.0, 69.0) \| 46.0  (24.0, 80.0) \| 51.0  (25.0, 70.0) \| 0.998 \| \| --- \| --- \| --- \| --- \| --- \| \| **Gender** \|  \|  \|  \| 1.000 \| \| Male \| 6 (60.0%) \| 6 (60.0%) \| 6 (60.0%) \|  \| \| Female \| 4 (40.0%) \| 4 (40.0%) \| 4 (40.0%) \|  \| \| **Race** \|  \|  \|  \| 0.126 \| \| Asian \| 1 (10.0%) \| 1 (10.0%) \| 0 (0.0%) \|  \| \| Black \| 2 (20.0%) \| 0 (0.0%) \| 2 (20.0%) \|  \| \| White \| 6 (60.0%) \| 3 (30.0%) \| 5 (50.0%) \|  \| \| Other/Multiracial \| 1 (10.0%) \| 1 (10.0%) \| 0 (0.0%) \|  \| \| Unknown/Declined \| 0 (0.0%) \| 5 (50.0%) \| 3 (30.0%) \|  \| \| **Ethnicity** \|  \|  \|  \| 0.322 \| \| Hispanic \| 3 (30.0%) \| 4 (40.0%) \| 3 (30.0%) \|  \| \| Non-Hispanic \| 7 (70.0%) \| 3 (30.0%) \| 5 (50.0%) \|  \| \| Unknown/Declined \| 0 (0.0%) \| 3 (30.0%) \| 2 (20.0%) \|  \| \| **Disease Severity** \|  \|  \|  \| 0.066 \| \| Asymptomatic/Mild \| 4 (40.0%) \| 5 (50.0%) \| 9 (90.0%) \|  \| \| Mild/Moderate \| 6 (60.0%) \| 5 (50.0%) \| 1 (10.0%) \|  \| \| **Number of Co-morbid Conditions** \|  \|  \|  \| 0.906 \| \| None \| 6 (60.0%) \| 5 (50.0%) \| 8 (80.0%) \|  \| \| One \| 2 (20.0%) \| 3 (30.0%) \| 1 (10.0%) \|  \| \| Two \| 1 (10.0%) \| 1 (10.0%) \| 0 (0.0%) \|  \| \| Three + \| 1 (10.0%) \| 1 (10.0%) \| 1 (10.0%) \|  \| \| **CDC week^b^ [end date]** at Visit 1 \| 32 [08Aug20]  (26 [27Jun20]**-** 41 [10Oct20]) \| 27.5 [11Jul20]  (24 [13Jun20]**-** 28 [11Jul20]) \| 32 [08Aug20]  (26 [27Jun20]**-** 41 [10Oct20]) \|  \| \| **Duration^a^**, days \|  \|  \|  \|  \| \| between Visit 1 - Visit 2 \| 7.0 (5.0, 12.0) \| 9.5 (4.0, 13.0) \| N/A \|  \| \| between Visit 2 - Visit 3 \| 7.5 (4.0, 20.0) \| 8.0 (4.0, 13.0) \| N/A \|  \| \| between Visit 3 - Visit 4 \| 7.0 \| 9.5 (7.0, 12.0) \| N/A \|  \| \| **N1 Ct value^a^** \|  \|  \|  \|  \| \| at Visit 1 \| 14.5 (9.8, 15.8) \| 34.1 (31.7, 36.3) \| N/A \|  \| \| at Visit 2 \| 26.6 (24.2, 33.9) \| 36.1 (30.8, 38.2) \| N/A \|  \| \| at Visit 3 \| 35.7 (32.4, 38.2) \| 35.6 (32.2, 38.5) \| N/A \|  \| \| at Visit 4 \| 34.3 \| 33.6 (33.6, 33.7) \| N/A \|  \| |

Abbreviations: Ct=cycle threshold, **^a^**Median (Min, Max)**^b^**Median (IQR) **or Median (Q1-Q3) or Median (25^th^ percentile-75^th^ percentile) or Median (lower quartile-upper quartile)**

Differences between groups were determined using the Kruskal-Wallis test for variables with non-parametric distribution and by Fisher’s Exact test for categorical variables. P-value <0.05 was considered significantly different between groups.
